# Photoelectrochemical Performance of Brookite Titanium Dioxide Electrodeposited on Graphene Foam for Portable Biosensors

**DOI:** 10.1021/acsomega.4c08624

**Published:** 2024-12-16

**Authors:** José L. Bott-Neto, Thiago S. Martins, Gabriel J. C. Pimentel, Osvaldo N. Oliveira, Frank Marken

**Affiliations:** † São Carlos Institute of Physics, University of São Paulo, São Carlos, São Paulo 13560-970, Brazil; ‡ Department of Chemistry, University of Bath, Claverton Down, Bath, England BA2 7AY, U.K.; ∥ Department of Chemistry, Molecular Sciences Research Hub, Imperial College London, 82 Wood Lane, London, England W12 0BZ, U.K.; § Brazilian Nanotechnology National Laboratory, Brazilian Center for Research in Energy and Materials, Campinas, São Paulo 13083-970, Brazil; ⊥ Institute of Chemistry, University of Campinas, Campinas, São Paulo 13083-970, Brazil

## Abstract

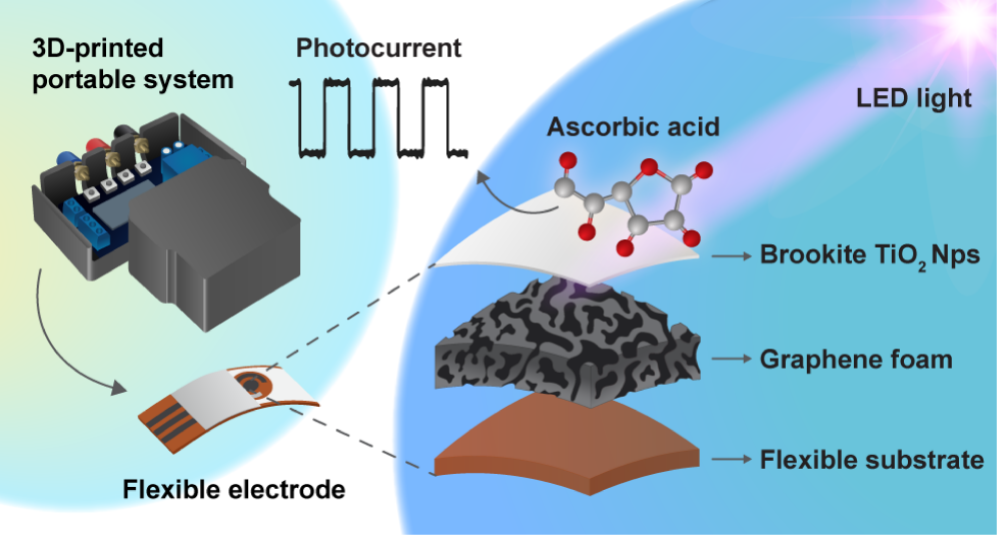

We discuss the photoelectroanalytical performance of a brookite-phase titanium dioxide (TiO_2_) platform electrodeposited onto graphene foam (GF) at low temperatures. The scalable electrosynthesis process eliminates the need for thermal annealing, which is impractical for carbon-based electrodes. Films resulting from a 10 min electrodeposition (TiO_2_-10/GF) exhibit enhanced photocurrents, reaching 170 μA cm^–2^_GEO_—twice the value for TiO_2_ films on traditional screen-printed carbon electrodes (82 μA cm^–2^_GEO_). The increased photocurrent density makes TiO_2_-10/GF ideal for on-site photoelectrochemical biosensors as it allows for the use of compact systems with low-power LEDs.

## 1 Introduction

Photoelectrochemical (PEC) sensors show potential for clinical diagnostics and environmental monitoring, offering low detection limits by minimizing background signals. This is possible owing to the separation between the readout source and the excitation source, which, in this case, is light.^1^ The miniaturization and cost reduction of these devices require the use of compact light sources, printed electrodes, and photoactive nanomaterials that operate with low-power irradiation.^2^ Titanium dioxide (TiO_2_) is used in PEC analysis due to its photoactivity, cost-effectiveness, photostability, biocompatibility, and low toxicity.^3^ TiO_2_ exists in three main crystal structures: anatase, which is stable at low temperatures; brookite, typically found in minerals but challenging to synthesize; and rutile, which is stable at higher temperatures.^4^

Platforms with enhanced photoactivity have been reported by combining TiO_2_ with graphene-based materials. These composites offer large specific surface areas and improved conductivity, making them ideal for photocatalysis applications. For example, reduced graphene oxide with TiO_2_ nanoparticles was used for photocatalytic degradation of the pollutant 4-nitrophenol in water.^5^ Graphene/TiO_2_ core–shell nanofibers with embedded graphene nanofibers were evaluated for phenol photodegradation.^6^ Few-layer graphene oxide encapsulated with TiO_2_ nanoparticles was used in the photocatalytic degradation of the organic water pollutant rhodamine B, with a 3-fold degradation rate compared with pure TiO_2_.^7^ Three-dimensional (3D) architectures, such as graphene foam (GF), are attractive for their conductive network and high porosity, which minimize steric hindrance to immobilize biomolecules with preserved activity,^8^ and improve photoelectrochemical performance.

The production of composites and reproducible films with TiO_2_ is challenging because of its low dispersibility. To address this limitation, we present a scalable method for synthesizing brookite on a graphene foam electrode (TiO_2_/GF) without the need for thermal annealing. Thermal annealing is commonly used to increase the crystallinity and improve the properties of semiconductor films.^9,10^ However, this process can be energy-intensive, especially for large-scale applications. In contrast, the method presented here eliminates the need for thermal annealing, potentially reducing energy consumption and simplifying the synthesis process. We compared the photoelectrochemical performance of TiO_2_/GF with that of laboratory-produced electrodes using carbon ink (CNPs) modified under the same conditions. The tests were conducted with 0.1 M ascorbic acid (AA) since it is being used extensively as a probe in photoelectrochemical immunosensors,^11^ aptasensors,^12^ and genosensors.^13^ The integration of a miniaturized, user-friendly, 3D-printed system with the TiO_2_/GF electrode demonstrates significant potential for on-site applications.

## 2 Experimental Section

### 2.1 Electrodes, Reagents, and Solutions

Graphene foam electrodes (GF; Gii-Sens) were purchased from Integrated Graphene LTD (Gii-Sens-40-Ag-AgCl-000050, Scotland). Printed carbon electrodes (PCEs) were manufactured according to the procedure described by Martins et al.^14^ A 20% (w/v) titanium(III) chloride solution was acquired from Thermo Scientific (England, United Kingdom). l-ascorbic acid (AA, ≥ 98%), potassium chloride (KCl, ≥ 99%), sodium bicarbonate (NaHCO_3_, ≥ 99.8%), sodium chloride (NaCl, ≥ 99%), sodium phosphate dibasic (Na_2_HPO_4_, ≥ 98%), potassium phosphate monobasic (KH_2_PO_4_, ≥ 99%), potassium hexacyanoferrate(II) trihydrate (K_4_[Fe(CN)_6_]·3H_2_O, 99%), and potassium hexacyanoferrate(III) (K_3_[Fe(CN)_6_], 99%) were obtained from Sigma-Aldrich (England, United Kingdom). The silver–silver chloride conductive ink used for the pseudoreference electrode (Ag|AgCl) was obtained from TICON (Sorocaba, Brazil). Ultrapure water, provided by a Thermo Fisher system, had a resistivity of 18.2 MΩ cm. The phosphate-buffered saline (PBS) solution was formulated at the following concentrations: 137 mM NaCl, 10 mM Na_2_HPO_4_, 1.8 mM KH_2_PO_4_, and 2.7 mM KCl.

### 2.2 Instrumentation

Raman spectroscopy was performed by using a Renishaw Qontor confocal Raman microscope with a 532 nm excitation wavelength. Scanning electron microscopy (SEM) images were obtained with a JEOL JSM-7900F microscope operating at an accelerating voltage of 5.0 kV. Electrochemical impedance spectroscopy (EIS) measurements were carried out using a CompactStat system from Ivium Technologies (The Netherlands), while all other electrochemical tests were conducted with a Metrohm Autolab potentiostat (model PGSTAT12).

### 2.3 Electrosynthesis of Brookite Titanium Dioxide

Brookite TiO_2_ was electrodeposited onto GF (or PCE) by using an electrochemical cell with temperature control, featuring a silver–silver chloride electrode (3.0 M KCl) as the reference and a printed carbon as the counter electrode. A 25 mM TiCl_3_ solution, adjusted to pH 2.5 and heated to 80 °C, was employed.^15^ Electrodeposition was carried out at 1.5 V for 10, 20, and 30 min, resulting in TiO_2_-10/GF (or TiO_2_-10/PCE), TiO_2_-20/GF, and TiO_2_-30/GF electrodes, respectively. The electrodes were then air-dried at room temperature. One carbon electrode was then coated with silver–silver chloride conductive ink to serve as a pseudoreference electrode (Ag|AgCl).

### 2.4 Electrochemical and Photoelectrochemical Measurements

**Scheme 1:**
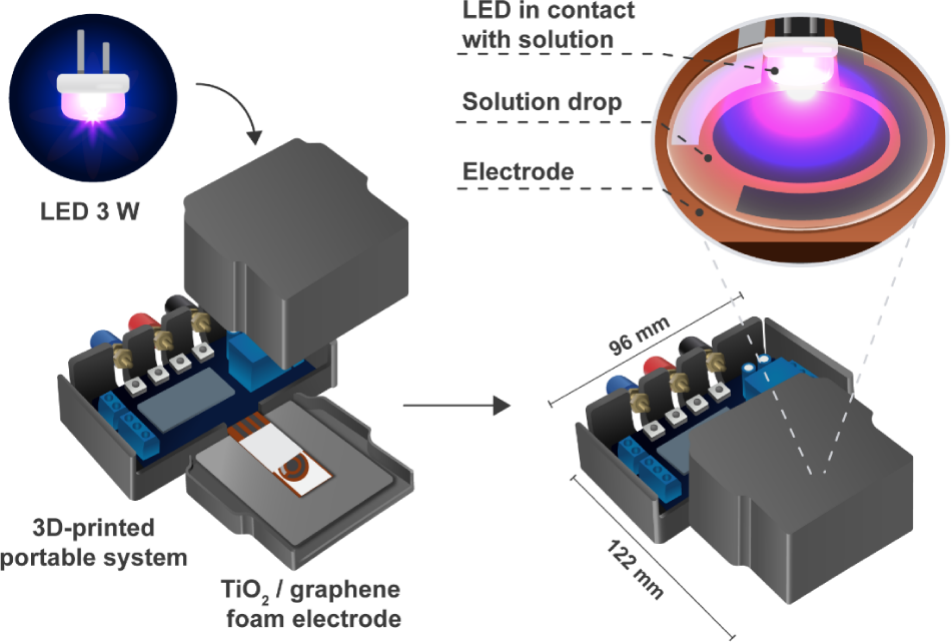
A 3D-Printed Portable Photoelectrochemical System Includes a 3 W LED, Connectors for the Reference Electrode (RE), Working Electrode (WE), and Counter Electrode (CE), and a TiO_2_/GF Electrode

Scheme 1 illustrates the 3D-printed portable photoelectrochemical system used for the photocurrent measurements, which includes a 3 W LED light (410 nm, 350 mW cm^–2^), a relay module to control the ON-OFF illumination cycles, and a cover to avoid external light interference. Further details can be found in our previous work.^1^ Transient current measurements were performed with a potential of 0 V vs the open-circuit potential (OCP). Linear sweep measurements used a potential range from −0.2 to 0.5 V versus Ag|AgCl at a scan rate of 2 mV s^–1^. ON-OFF cycles of 20 s for transient current curves, 10 s for linear sweep, and 60 s for the OCP measurements were adopted. The photoelectrochemical experiments were conducted in a PBS solution containing 0.1 M AA. EIS was performed with a 5 mM solution of [Fe(CN)_6_]^3–^/^4–^ (containing 5 mM K_4_[Fe(CN)_6_] and 5 mM K_3_[Fe(CN)_6_]) in 0.1 M KCl, from 1 Hz to 10 kHz with a 0 V bias versus OCP. All experiments were conducted with a 100 μL solution volume.

## 3 Results and Discussion

### 3.1 Characterization

**Figure 1:**
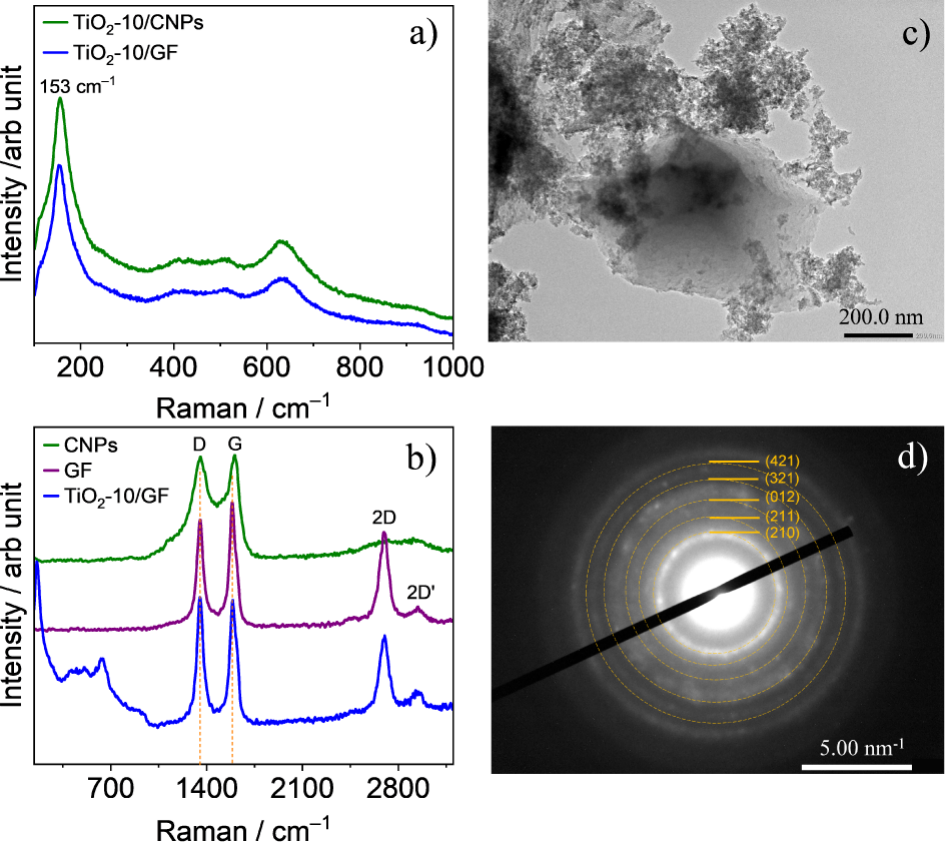
Raman spectra of different electrode materials: (a) TiO_2_ electrodeposited for 10 min on carbon (TiO_2_-10/CNPs) and on graphene foam (TiO_2_-10/GF) between 100 and 1000 cm^–1^; (b) CNPs, GF, and TiO_2_-10/GF electrodes from 140 to 3200 cm^–1^. (c) TEM image and (d) electron diffraction images of TiO_2_-10/GF.

The Raman spectra for the TiO_2_-10/GF and TiO_2_-10/CNPs electrodes in Figure 1a show bands at 153, 252, 322, 412, and 633 cm^–1^, characteristic of brookite TiO_2_.^16−18^ For graphene foam, the Raman spectra display bands at 1352, 1588, 2693, and 2942 cm^–1^, associated with D, G, 2D, and 2D’ vibrational modes (Figure 1b). The D peak corresponds to the disordered structure of carbon black (amorphous carbon), while the G peak is associated with the high-frequency vibration of the carbon network. The D and 2D’ peaks are attributed to the interactions between two layers of graphene and disordered graphene/nanographene, respectively.^19^ The TEM image in Figure 1c for TiO_2_/GF reveals multilayer graphene structures decorated with TiO_2_ nanoparticles. Electron diffraction analyses confirm the brookite phase, as shown in Figure 1d.

**Figure 2:**
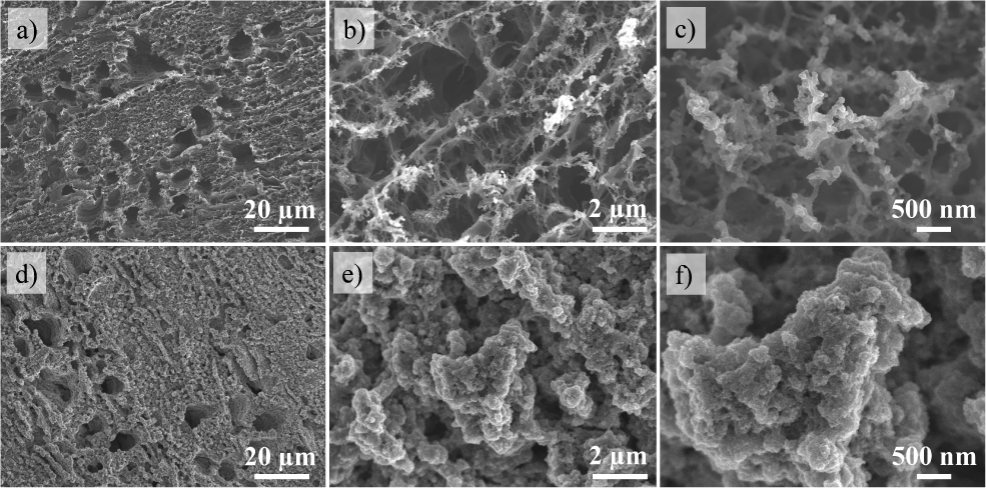
SEM images of (a-c) graphene foam (GF) and (d-f) TiO_2_ electrodeposited for 10 min on graphene foam (TiO_2_-10/GF) electrodes at magnifications of 1.00 kx (a, d), 5.00 kx (b, e), and 25.00 kx (c and f).

**Figure 3:**
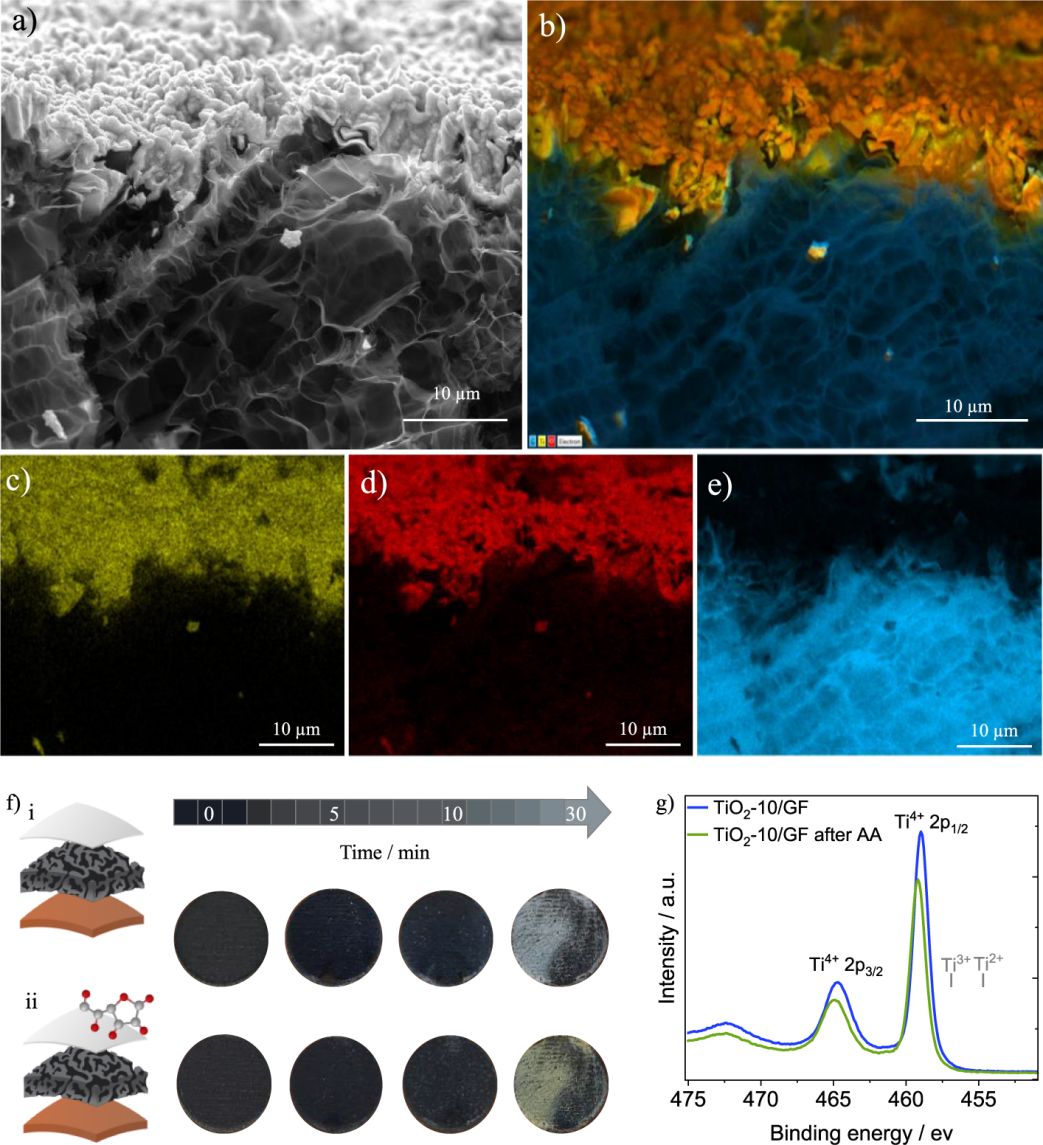
(a) Cross-sectional SEM images of the TiO_2_ electrodeposited for 10 min on graphene foam (TiO_2_-10/GF) electrode. (b) EDS mapping of the TiO_2_-10/GF elements showing (c) Ti, (d) O and (e) C distribution. (f-i) Images of graphene foam (GF), TiO_2_ electrodeposited for 5 (TiO_2_-5/GF), 10 (TiO_2_-10/GF) and 30 min (TiO_2_-30/GF) before exposure to ascorbic acid (AA). (f-ii) Images of the same electrodes after 5 min of exposure to 0.1 M AA. (g) XPS spectra of the TiO_2_-10/GF electrode before and after exposure to AA.

The SEM images in Figure 2a–c show that the GF electrode exhibits an interconnected microporous network, enabling electrolyte ions to penetrate into the graphene electrode.^20^ In contrast, the CNPs electrode, composed of graphite and carbon nanoparticles, has a more compact surface (Figure S1a). Figure 2d–f shows that the GF electrode retains a significantly larger surface area compared to the CNPs electrode, even after TiO_2_ electrodeposition (Figures 2e and S1). The cross-sectional images and EDS mapping in Figure 3a–e show the TiO_2_-10/GF and GF electrodes. The graphene foam electrodes have a carbon layer 37.5 ± 2.5 μm thick and a TiO_2_ layer 4.8 ± 0.8 μm thick (Figure S2). This TiO_2_ layer is 2.8 times thicker than the electrodeposited TiO_2_ on CNPs (1.7 μm), likely due to better penetration of TiO_2_ into the porous graphene structure. However, TiO_2_ particles primarily form on the top surface rather than within the GF film, as evidenced by the mapping images (Figure 3c–e).

Figure 3f(i) shows the GF, TiO_2_-5/GF, TiO_2_-10/GF, and TiO_2_-30/GF electrodes before exposure to AA, emphasizing the impact of different electrodeposition times on the TiO_2_ layer, while Figure 3f(ii) shows the same electrodes after 5 min of exposure to 0.1 M AA. The amount of electrodeposited material increases with time. Electrodes prepared for up to 10 min have a uniform film, while 30 min of TiO_2_ deposition results in a nonuniform coating. After interaction with the AA solution, all TiO_2_/GF electrodes exhibited a color change from gray to yellow, indicating that electrons in the conduction band are altering the reflected light^21^ (vide infra). The XPS spectra of TiO_2_-10/GF before and after exposure to AA are shown in Figure 3g. The pristine sample exhibits two peaks at 464.73 and 458.98 eV, consistent with the Ti^4+^ oxidation state.^22−24^ Following acid exposure, a shift to higher binding energies (464.93 and 459.20 eV) is observed, suggesting charge transfer from the AA ligand to the TiO_2_ conduction band.

### 3.2 Electrochemical Characterization

**Figure 4:**
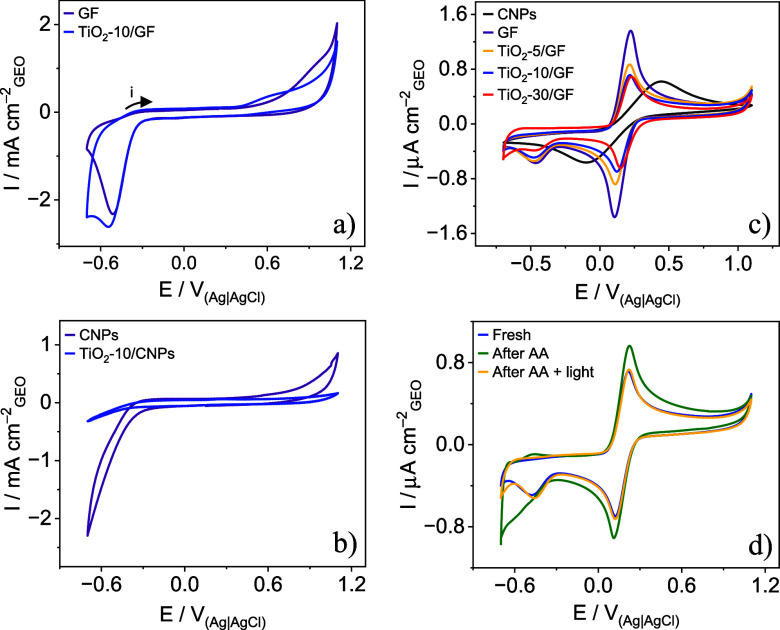
Cyclic voltammograms recorded at 50 mV s^–1^ in PBS (pH 7.4) for electrodes: (a) carbon (CNPs) and TiO_2_ electrodeposited for 10 min on CNPs (TiO_2_-10/CNPs), and (b) graphene foam (GF) and TiO_2_ electrodeposited on graphene foam (TiO_2_-10/GF). Cyclic voltammograms were recorded at 50 mV s^–1^ in 0.1 M KCl with 5 mM [Fe(CN)_6_]^3–/4–^ for (c) CNP, GF, TiO_2_-5/GF, TiO_2_-10/GF, and TiO_2_-30/GF, and (d) fresh TiO_2_-10/GF, TiO_2_-10/GF after exposure to AA, and TiO_2_-10/GF after exposure to AA under irradiation with a 3 W LED light (410 nm).

Cyclic voltammograms were recorded in a PBS solution (pH 7.4) at 50 mV s^–1^ for CNPS, GF, TiO_2_-10/CNPs, and TiO_2_-10/GF electrodes. As shown in Figure 4a, the background current of graphene foam remains mostly unchanged after TiO_2_ electrodeposition, a similar observation being made for the carbon substrate in Figure 4b. A prominent reduction peak at −0.5 V can be assigned to oxygen adsorbed onto GF.^25^ The carbon oxidation potentials are very close: 0.45 V for GF and 0.44 V for CNPs. The currents associated with carbon oxidation and water oxidation (potentials above 1.0 V) are higher for GF, most likely because of its larger surface area, as confirmed by the SEM images.

To assess the electrochemically active surface area, voltammograms were obtained in a 0.1 M KCl solution containing 5 mM [Fe(CN)_6_]^3–/4–^. Figure 4c shows more reversible redox pairs for GF than for CNPs electrodes, with a potential difference (Δ*E*) of 0.12 V for GF (calculated as Ep_a_ – Ep_c_) and 0.53 V for CNPs. Here, “Ep_a_” represents the anodic peak potential, and “Ep_c_” denotes the cathodic peak potential. The anodic peak current (Ip_a_) and cathodic peak current (Ip_c_) are both 1.36 μA cm^–2^ for GF, whereas for CNPs, Ip_a_ is 0.63 μA cm^–2^ and Ip_c_ is −0.56 μA cm^–2^. These values indicate that the electrochemically active surface area of GF is 2.3 times that of CNPs, as inferred from the Randles-Sevcik method. Moreover, the voltammograms show a decrease in peak current intensity as the electrodeposition time increases from 5 to 30 min.

**Scheme 2:**
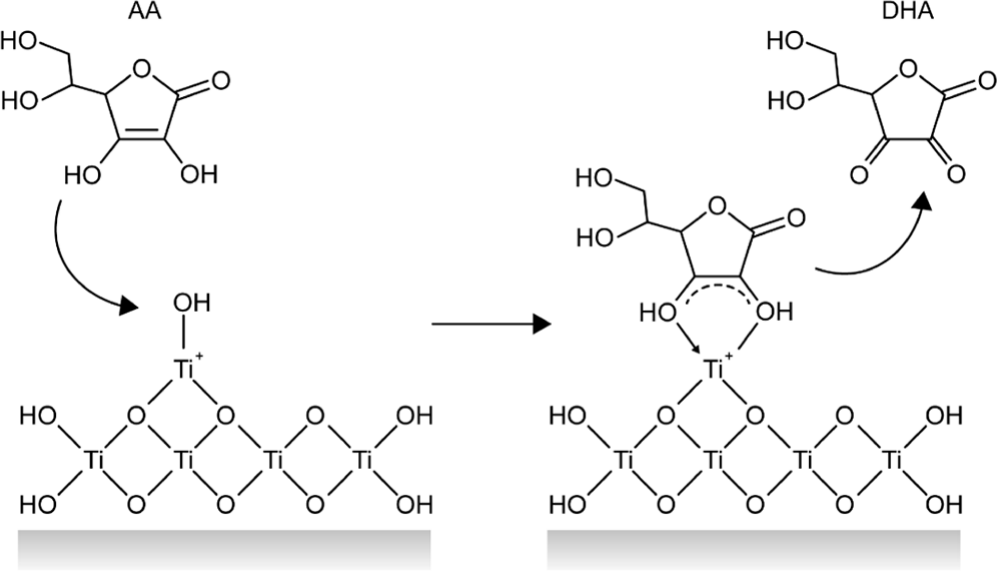
Interaction of Ascorbic Acid (AA) with the TiO_2_ Surface On the left, AA interacts with a hydroxylated Ti^4+^ site, while on the right, AA is oxidized to dehydroascorbic acid (DHA).

Further characterization of the electrodes after exposure to AA, shown in Figure 4d, revealed oxidation and reduction peaks in the −0.7 to 0.3 V range. The TiO_2_ surface has Ti atoms with incomplete coordination, making them highly reactive.^26^ These Ti atoms form charge transfer (CT) complexes with electron-donating ligands, causing a red shift in absorption.^26−28^ The XPS spectra of TiO_2_-10/GF before and after exposure to AA, shown in Figure 3g, corroborate this observation. Scheme 2 illustrates the mechanism where AA is oxidized to dehydroascorbic acid (DHA) and subsequently desorbs from the electrode surface,^29^ as evidenced by the disappearance of peaks in the cyclic voltammogram.

**Figure 5:**
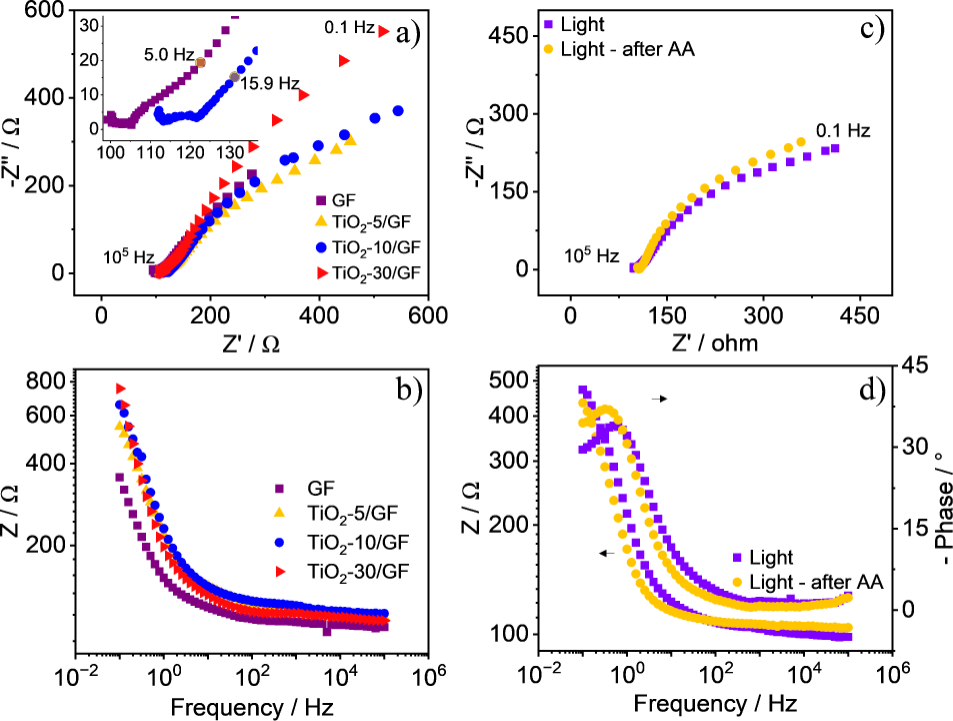
EIS spectra in a 0.1 M KCl solution containing 5 mM [Fe(CN)_6_]^3–^/^4–^. (a) Nyquist and (b) Bode plots for graphene foam (GF), TiO_2_ electrodeposited on GF for 5 (TiO_2_-5/GF), 10 (TiO_2_-10/GF), and 30 min (TiO_2_-30/GF) electrodes. (c) Nyquist and (d) Bode plots of the TiO_2_-10/GF electrode under LED light irradiation, before and after exposure to AA solution.

Figure 5 presents the EIS spectra obtained in a 0.1 M KCl solution containing 5 mM [Fe(CN)6]^3–^/^4–^ for the GF, TiO_2_-5/GF, TiO_2_-10/GF, and TiO_2_-30/GF electrodes. The Nyquist plots in Figure 5a display a small semicircle at high frequencies, indicating kinetic control of the charge transfer process, and a linear region at low frequencies, representing diffusional control of the electroactive species. The GF electrode exhibits an incomplete semicircle, whereas the TiO_2_-10/GF electrode shows a more defined semicircular pattern, as shown in the inset of Figure 5a. This pattern is characteristic of high surface area electrodes, where increased capacitance can lead to distortion or disruption of the semicircular shape in the Nyquist plot.^30^ In addition, the electrodeposition of TiO_2_ does not enhance the charge transfer resistance significantly, which is the behavior expected for semiconductors. This is attributable to the electrode’s high porosity and easy access of the [Fe(CN)_6_]^3–^/^4–^ redox probe to the conductive graphene surface. The ohmic resistance of the TiO_2_-5/GF and TiO_2_-10/GF electrodes (116 and 121 Ω, respectively) is slightly increased compared to the GF electrode (105 Ω), likely resulting from TiO_2_ accumulation on the GF surface. However, the resistance decreases to 112 Ω for the TiO_2_-30/GF electrode. The Bode plot in Figure 5b shows an increase in impedance at low frequencies with longer electrodeposition times. This is attributed to the formation of a thicker diffusion layer, which reduces the available surface area for the TiO_2_ deposition.

EIS analyses of the TiO_2_-10/GF electrode were conducted before and after exposure to an AA solution under light irradiation. The Nyquist plots in Figure 5c show an increase in the ohmic resistance. The Bode plot in Figure 5d reveals a decrease in the total impedance and a shift of the maximum frequency (*f*_max_) to lower values. This shift in *f*_max_, which is related to the electron lifetime (τ_e_) in the material through the equation τ_e_ = 1/(2π*f*_max_),^31,32^ indicates an increased electron lifetime. A lower *f*_max_ suggests more time for electrons to participate in chemical reactions before recombining. The increased photocatalytic current intensity, resulting from a reduced rate of charge carrier recombination, supports this observation. Thus, the use of AA enhances the photocatalytic efficiency of the TiO_2_-10/GF electrode, making it an attractive probe for developing advanced immunosensors, aptasensors, and genosensors.

### 3.3 Photoelectrochemical Properties

**Figure 6:**
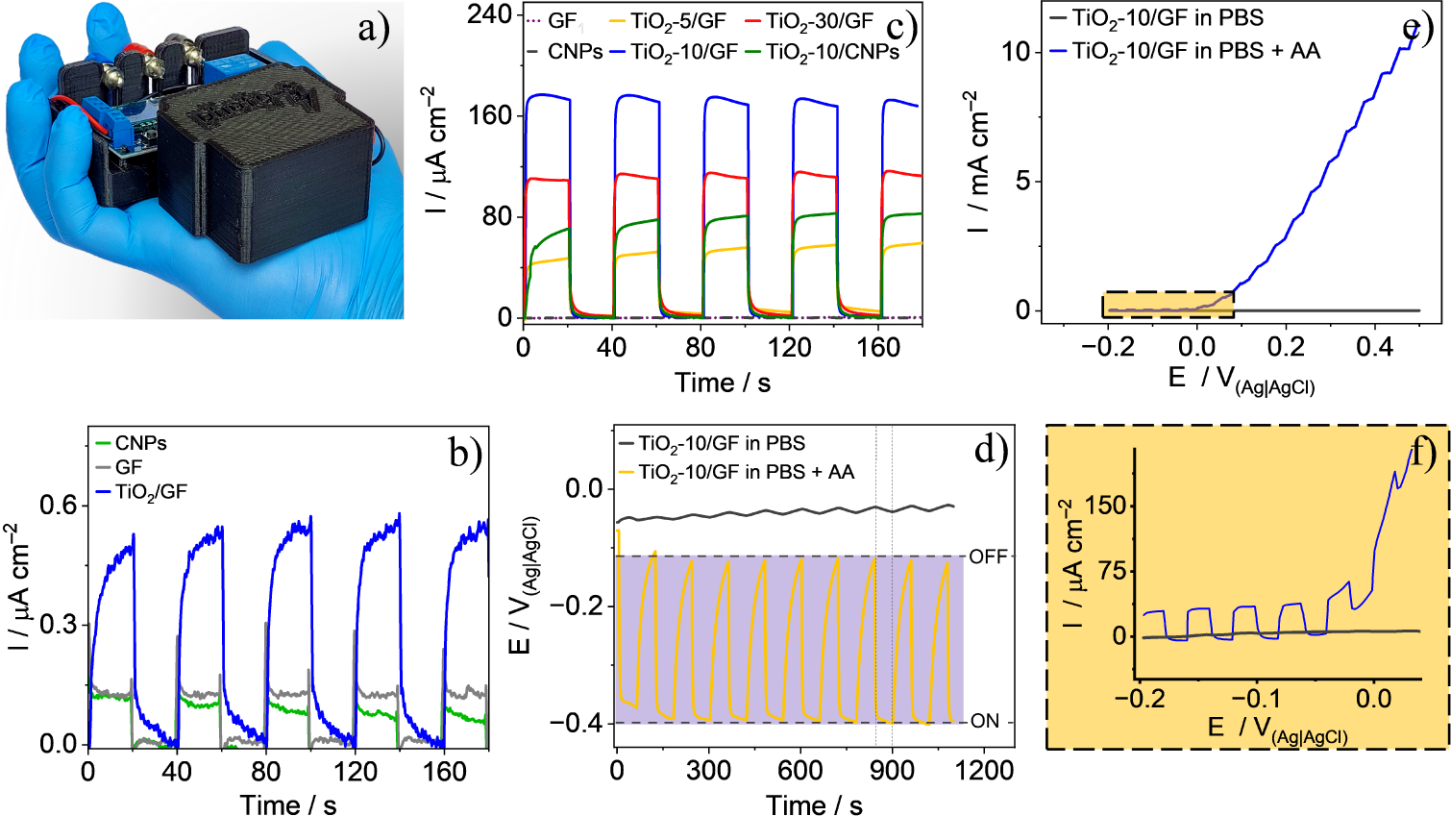
(a) Photo of the 3D-printed portable photoelectrochemical system used in the measurements. Transient current curves in PBS solution (pH 7.4) at 0 V vs OCP (∼0 V vs Ag|AgCl) under 410 nm LED light (20 s ON/20 s OFF) (b) without and (c) with 0.1 M ascorbic acid (AA), for different materials. (d) OCP measurements (60 s ON and OFF). (e–f) Linear sweep voltammetry without and with 0.1 M AA (10 s ON/10 s OFF).

The photo of the system used for photocurrent measurements is shown in Figure 6a. Figure 6b displays the transient current curves obtained in 0.1 M PBS solution under visible LED light irradiation (410 nm) for the CNPs, GF, and TiO_2_-10/GF electrodes. The photocurrents were 0.04, 0.12, and 0.52 μA cm^–2^ for the CNPs, GF, and TiO_2_-10/GF electrodes, respectively. Figure 6c shows the curves in the presence of a 0.1 M AA solution. The GF and CNPs electrodes do not show a significant increase in the photocurrent when AA is added. In contrast, the TiO_2_-modified electrodes exhibit a boost in photocurrent, which can be attributed to the reduction in charge carrier recombination, as discussed in the previous section. The photocurrents for TiO_2_-5/GF, TiO_2_-10/GF, TiO_2_-30/GF, and TiO_2_/CNPs are 58.0, 170.4, 114.4, and 82.0 μA cm^–2^, respectively.

Since the applied potential can affect the stability and selectivity in photoelectrochemical measurements, mainly due to the contribution of faradaic current, the OCP was studied in the presence and absence of 0.1 M AA. Figure 6d shows the OCP values for GF and TiO_2_-10/GF electrodes with and without AA (in PBS solution) during 60-s ON/OFF cycles. The potential changes are minimal without AA for the TiO_2_-10/GF electrode. With AA, the OCP ranges from −0.11 V (dark) to −0.39 V (light). Initially, the OCP values in the presence and absence of AA are close, but they do not return to the starting potential after the cycles begin. Attempts to extend the cycle times led to solution evaporation caused by heat from prolonged LED activation, an issue not seen in shorter cycles.

Figure 6e,f show that the electrochemical oxidation of AA begins just after −0.05 V. Experiments were consistently performed at OCP values from −0.05 to −0.07 V. No photocurrent gain is observed when increasing the potential from −0.2 to −0.05 V. The increase in potential only raises the current attributable to faradaic processes, with similar photocurrent at both high and low potentials. Therefore, performing measurements at the equilibrium potential helps one to achieve a lower baseline and avoids interference from the electrooxidation of AA and organic compounds in the sample.

## 4 Conclusion

This work presents the electrosynthesis of a photoactive TiO_2_ phase on graphene foam electrodes without the need for thermal annealing. The low-temperature electrodeposition method partially embeds TiO_2_ into the porous graphene foam, resulting in a photocurrent of 170 μA cm^–2^_GEO_-approximately 2.1 times the value for traditional carbon-based printed electrodes (82 μA cm^–2^_GEO_). TiO_2_-10/GF outperforms TiO_2_/CNPs electrodes, demonstrating that graphene foam enhances photocurrents and holds promise for TiO_2_-based photoelectrochemical platforms. Although TiO_2_ was successfully electrodeposited into the graphene foam film, only the top layer was effectively modified (due to nucleation and growth at the surface). One may expect enhanced photoanodes if TiO_2_ can be incorporated deeper into the foam structure. This is challenging and will require further work. Our findings also support the development of biosensors utilizing AA as a probe in conjunction with compact, low-power visible light sources, making the device suitable for point-of-care applications.

## Data Availability

The data supporting this article have been included as part of the Supporting Information.

## Supporting Information Available

SEM image of CNPs before and after TiO_2_ electrodeposition. Cross-sectional SEM of GF and TiO_2_-10/GF (PDF)
