# Supplementary material for: Photoelectrochemical Performance of Brookite Titanium Dioxide Electrodeposited on Graphene Foam for Portable Biosensors
Source: ACS Omega. 2024 Dec 16;9(52):51474–80. doi: 10.1021/acsomega.4c08624 (PMC11696410; doi:10.1021/acsomega.4c08624)
Supplement: Supplementary file 1 — ao4c08624_si_001.pdf [file ao4c08624_si_001.pdf]

Supplementary information

Photoelectrochemical Performance of Brookite Titanium Dioxide Electrodeposited on Graphene Foam for Portable Biosensors

José L. Bott-Neto[a,b,d], Thiago S. Martins[a,c,*], Gabriel J. C. Pimentel[d,e], Osvaldo N. Oliveira Jr[a], and Frank Marken[b]*

[a]São Carlos Institute of Physics, University of São Paulo, 13560-970 São Carlos, SP, Brazil

[b]Department of Chemistry, University of Bath, Claverton Down, Bath BA2 7AY, England, UK

[c]Department of Chemistry, Molecular Sciences Research Hub, Imperial College London, 82 Wood Lane, London W12 0BZ, England UK

[d]Brazilian Nanotechnology National Laboratory, Brazilian Center for Research in Energy and Materials, Campinas, São Paulo13083-970, Brazil

[e]Institute of Chemistry, University of Campinas, Campinas, São Paulo 13083-970, Brazil

*Corresponding authors. E-mail: joseluiz.bott@gmail.com; thiagoserafimartins@gmail.com

Figure S1. SEM image of the working electrode for the CNPs electrode (a) before and (b) after TiO_2 electrodeposition (time = 10 min).

Figure S2. Cross-sectional SEM images of the working electrode region: (a) GF and (b) TiO2-10/GF.

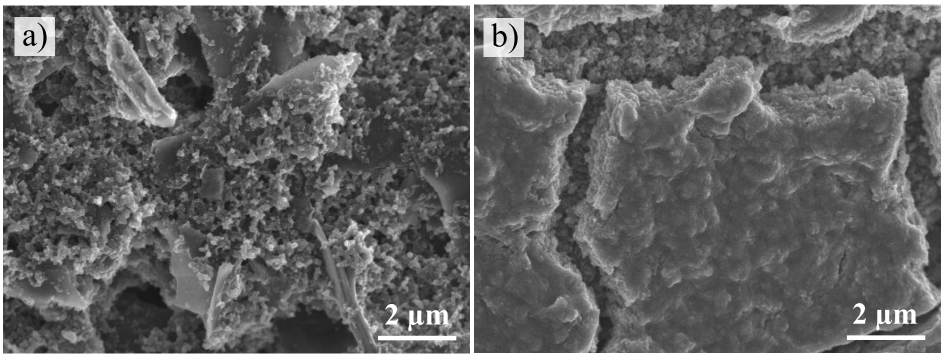

Figure S1. SEM image of the working electrode for the CNPs electrode (a) before and (b) after TiO_2 electrodeposition (time = 10 min).

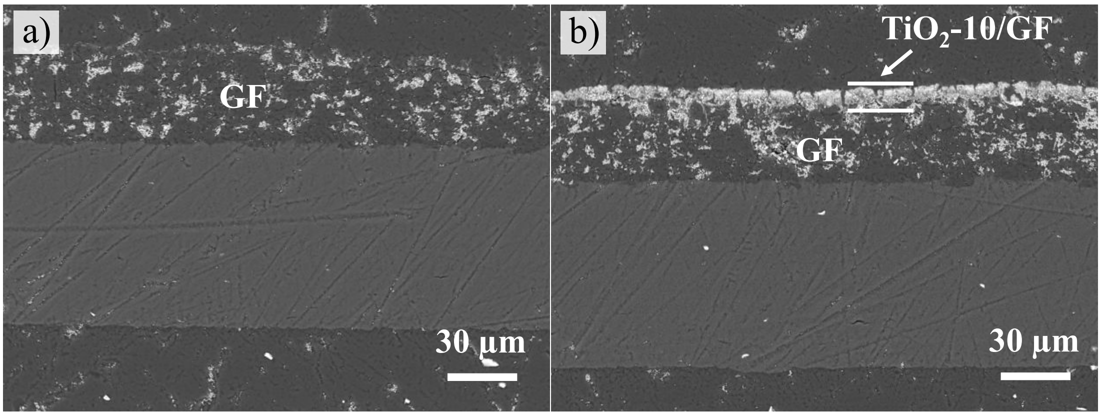

Figure S2. Cross-sectional SEM images of the working electrode region: (a) GF and (b) TiO2-10/GF.
